# Sequencing, *de novo* assembly and comparative analysis of *Raphanus sativus* transcriptome

**DOI:** 10.3389/fpls.2015.00198

**Published:** 2015-04-01

**Authors:** Gang Wu, Libin Zhang, Yongtai Yin, Jiangsheng Wu, Longjiang Yu, Yanhong Zhou, Maoteng Li

**Affiliations:** ^1^School of Life Science and Technology, Huazhong University of Science and TechnologyWuhan, China; ^2^National Key Lab of Crop Genetic Improvement, Huazhong Agricultural UniversityWuhan, China

**Keywords:** *Raphanus sativus*, transcriptome, RNA sequencing, transcription factor, simple sequence repeats

## Abstract

*Raphanus sativus* is an important *Brassicaceae* plant and also an edible vegetable with great economic value. However, currently there is not enough transcriptome information of *R. sativus* tissues, which impedes further functional genomics research on *R. sativus*. In this study, RNA-seq technology was employed to characterize the transcriptome of leaf tissues. Approximately 70 million clean pair-end reads were obtained and used for *de novo* assembly by Trinity program, which generated 68,086 unigenes with an average length of 576 bp. All the unigenes were annotated against GO and KEGG databases. In the meanwhile, we merged leaf sequencing data with existing root sequencing data and obtained better *de novo* assembly of *R. sativus* using Oases program. Accordingly, potential simple sequence repeats (SSRs), transcription factors (TFs) and enzyme codes were identified in *R. sativus*. Additionally, we detected a total of 3563 significantly differentially expressed genes (DEGs, *P* = 0.05) and tissue-specific biological processes between leaf and root tissues. Furthermore, a TFs-based regulation network was constructed using Cytoscape software. Taken together, these results not only provide a comprehensive genomic resource of *R. sativus* but also shed light on functional genomic and proteomic research on *R. sativus* in the future.

## Introduction

As an important *Brassicaceae* plant with great economic value, *Raphanus sativus* was widely cultivated throughout the world. It was also reported that *R. sativus* has been used as a medicinal plant due to its exceptional medicinal properties (Watt and Breyer-Brandwijk, 1962). For instance, the radish roots contain various peroxidases and could be used for medicinal purposes (Wang et al., 2002). With the development of next-generation sequencing technology, genetic information of *R. sativus* was gradually revealed in the recent years (Wang et al., 2013a; Xu et al., 2013; Zhang et al., 2013). For example, the transcriptome profiles of leaf tissue of *R. sativus* were analyzed by RNA-seq technology in our previous study. However, only 22 million clean reads were used and the unigenes generated in this study only represents a small proportion of *R. sativus* transcriptome because the sequencing coverage was simply not deep enough (Zhang et al., 2013). Therefore, there is an urgent need to analyze more transcriptome data of *R. sativus* tissues, which will facilitate their potential use as general reference for other related *R. sativus* studies.

Next-generation sequencing technology has provided a novel method both for gene mapping and transcriptome analysis (Schuster, 2008; Shendure and Ji, 2008; Wang et al., 2009). RNA-seq is not dependent on prior information of the genomic sequence of the target species, which has been widely applied for transcriptome-related studies in many *Brassicaceae* plant species (Paritosh et al., 2013; Wang et al., 2013b; Kim et al., 2014; Mudalkar et al., 2014). The *de novo* assembly of sequencing reads is an important step to obtain genome information, such as novel gene discovery, transcription factor (TF) discovery, Simple Sequence Repeat (SSR) mining, and gene expression profile analysis (Powell et al., 1996; Bouché et al., 2002; Heim et al., 2003; Jiao et al., 2003; Knowles and McLysaght, 2009; Zhang et al., 2012b). For example, it has been reported that 30 TF families containing approximately 1500 potential TFs were identified after the completion of the *Arabidopsis thaliana* genome sequencing project (Riechmann et al., 2000; Mitsuda and Ohme-Takagi, 2009). Also, SSRs are abundantly applied in molecular mapping, genetic diversity research, cultivar fingerprinting due to multiple advantages including co-dominant inheritance nature, high variability, high feasibility of detection, and good transferability (Li et al., 2004). Next-generation sequencing technology for SSR mining has been used in various plant species such as rubber tree (Li et al., 2012), castor bean (Qiu et al., 2010), sesame (Zhang et al., 2012a), and some *Brassicaceae* plants (Wang et al., 2007; Yamane et al., 2009; Kamei et al., 2010; Ohsako et al., 2010). However, so far, little has been reported about the TF and SSR profiles due to limited genome information in *R. sativus*.

In this study, we applied the RNA-Seq technology to deeply sequence the transcriptome of leaf tissue in *R. sativus*. A total of 70 million clean pair-end reads were obtained and used for the *de novo* assembly of transcriptome of *R. sativus*. The sequencing coverage was comprehensive enough to identify most unigenes and major metabolic pathways. The *de novo* assembly obtained a total of 68,086 unigenes with an average length of 576 bp. We further downloaded root sequencing data (SRX256970 and SRX263753) and merged the clean reads of leaf and root sequencing data for *de novo* assembly using Trinity and Oases programs. The Oases-assembled unigenes were used for functional annotation and downstream analyses. Furthermore, 31,875 potential SSRs with different motifs were detected. Finally, a large number of transcription factors and enzymes were identified by comparing with available databases. These results provide a comprehensive genomic resource for gene expression analysis and functional genomics research on *R. sativus* in the future.

## Materials and methods

### Plant material and transcriptome sequencing

The *R. sativus* big root radish (No specific permissions were required to use this *R. sativus* species) was provided by Prof. Jiangsheng Wu of Huazhong Agricultural University, which was cultivated in the experimental field of Huazhong University of Science and Technology in the year of 2012 and 2013. The method for total RNA isolation of leaf tissues was according to the methods that described earlier (Zhang et al., 2013). The isolated RNAs were prepared for the construction of RNA-seq libraries. In short, the mRNAs collected from leaf tissues of *R. sativus* were first fragmented and further used to synthesize first-strand cDNAs with hexamer and reverse transcriptase (Promega). Subsequently, the second-strand cDNAs were synthesized with DNA polymerase I and RNase H. The obtained cDNA fragments were then purified, end-repaired, A-tailed, and ligated to index adapters (Illumina). The ligation products were amplified by PCR and sequenced using the Illumina GAIIx and a 100 bp pair-end sequencing protocol was employed.

### *De novo* transcriptome assembly of *R. sativus*

Raw reads were initially processed by Illumina Pipeline Software to get sequence information. Then the reads were cleaned by removing adaptor sequences and low quality reads with ambiguous sequences “N.” The Trinity program (Grabherr et al., 2011) was carried out to assemble the clean reads to obtain non-redundant unigenes. Clean reads with a certain overlaps were combined to form longer contiguous sequences (contigs), which were joined into scaffolds that were further assembled by gap filling to acquire unigenes. Transcriptome coverage is highly variable owing to differential gene expression in cells, so there is no single absolutely optimal k-mer length for transcriptome assembly. In this study, 25 was set as the default k-mer size for the *de novo* assembly of the transcriptome, while other parameters used default values. The length of the assembled unigenes for further study should be no less than 200. For Oases assembly, Velvet was first run using k-mer length of 31 along with other default parameters. The contigs produced by Velvet were post-processed using Oases with k-mer of 31.

### Transcriptome annotation of *R. sativus*

All assembled unigenes were compared with the NCBI non-redundant protein (Nr), Swissprot, Pfam and Trembl databases by using blastx with a typical cut-off *E*-value of 1 e^−5^ to search for homologs. Based on the results of the Nr database annotation, BLAST2GO (Conesa et al., 2005) was used to obtain Gene Ontology (GO) annotation of assembled unigenes for describing cellular component, molecular function, and biological process. WEGO (Ye et al., 2006) was employed to perform the GO functional classification for understanding the distribution of gene functions at the macro level. The unigenes were also searched against the Cluster of Orthologous Groups (COG) database to classify their functions. The KEGG (Kyoto Encyclopedia of Genes and Genomes) pathway of the assembled unigenes was annotated by mapping the sequences obtained from BLAST2GO to the contents of the KEGG metabolic pathway database (http://david.abcc.ncifcrf.gov/).

### Identification of differentially expressed genes in the leaf and root transcriptomes

Reads Per kb per Million reads (RPKM) was used to calculate gene expression level (Mortazavi et al., 2008). The differentially expressed genes (DEGs) was determined with a log-fold expression change (log FC) greater than 2 or less than −2 using a threshold of false discovery rates (FDR < 0.001) and a greater statistically significant value (*P* < 0.005).

### SSR mining of *R. sativus*

The SSR mining was performed following the method described previously (Zhang et al., 2012a). In this study, the SSRs were considered to include with five motifs: dinucleotide, trinucleotide, tetranucleotide, pentanucleotide, and hexanucleotide. For each motif of SSR, the number of contiguous repeat units was more than five. Statistical analysis was performed to summarize the number of SSRs with each type of motif and the length distribution of repeat units.

### Identification of transcription factors of *R. sativus*

In order to identify the transcription factors represented in *R. sativus* transcriptome, all assembled unigenes were searched against the plant transcription factor database (PlnTFDB; http://plntfdb.bio.uni-potsdam.de/v3.0/downloads.php) by using blaxtx with a cut-off *E*-value of 1 e^−5^ (Kalra et al., 2013).

### Detection of enzymes of *R. sativus*

For predicting the potential enzymes in the unigenes, the transcripts were probed against all the enzyme protein sequences at Enzyme nomenclature database (ENZYME; http://enzyme.expasy.org/) by using blaxtx with a cut-off *E*-value of 1 e^−5^ (Kalra et al., 2013).

### Transposable element analysis

We run RepeatMasker (http://www.repeatmasker.org/) with options “quick search” and species “Arabidopsis” to identify repetitive and transposable elements (TE) in *R. sativus* transcriptome. RepeatMasker version open-3.2.9, rmblastn version (1.2) 2.2.23 and RepBase update 20090604 were used in thisstudy.

## Results and discussion

### RNA-seq and *de novo* transcriptome assembly of *R. sativus*

**Figure 1:**
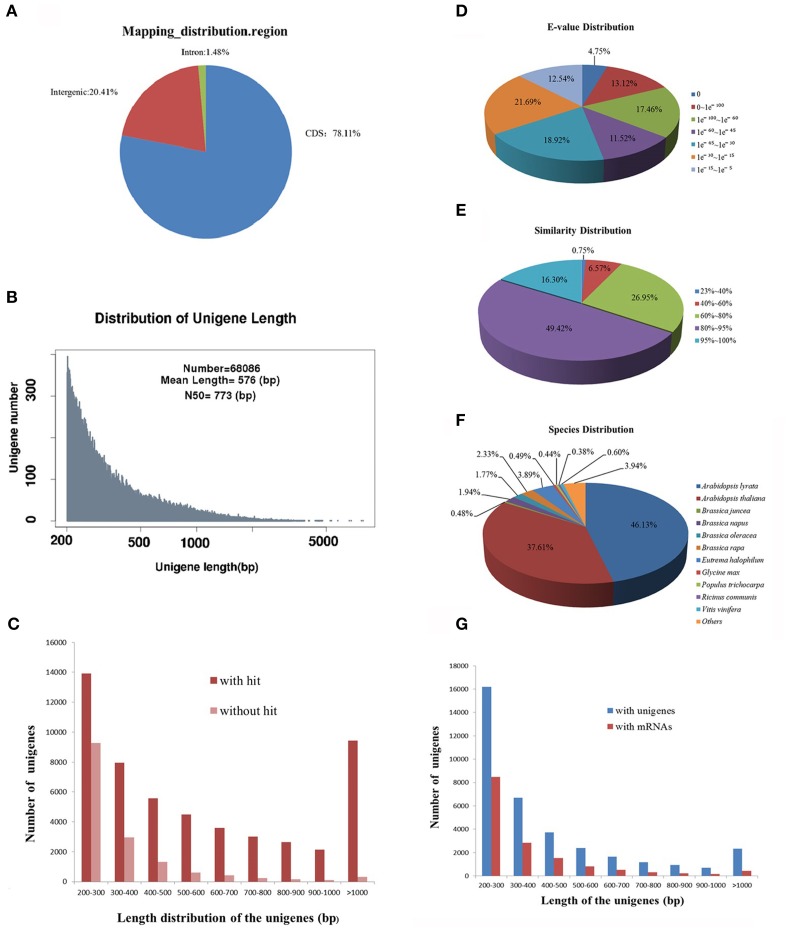
**Sequencing, *de novo* assembly and annotation of leaf transcriptome of *R. sativus* transcriptome. (A)** The clean reads distribution map of leaf tissue on *B. rapa* genome. **(B)** The *de novo* assembly result of leaf transcriptome. **(C)** Comparative analysis of unigenes length with or without hit against Nr and Swissprot databases. **(D)** *E*-value distribution of BLAST hits for each unique sequence against the Nr database. **(E)** Similarity distribution of the top BLAST hits for each sequence against the Nr database. **(F)** Species distribution is shown as a percentage of the total homologous sequences against the Nr database. **(G)** Comparative analysis of the leaf unigenes with the unigene and mRNA databases of *R. sativus*.

Next generation sequencing technology has been widely applied to characterize the transcriptome profiles in multiple non-model plants. Our previous study has analyzed the transcriptome of leaf tissues in *R. sativus* by RNA sequencing (Zhang et al., 2013). However, due to the low coverage of sequencing reads, this study had been not portrayed as a representative transcriptome assembly project but rather a survey of leaf tissues transcripts. Therefore, to obtain highly reliable *de novo* transcriptome assembly of leaf tissues in *R. sativus*, we reconstructed a cDNA library and performed transcriptome sequencing using Illumina sequencing platform. Initially, the base quality of the sequenced reads was evaluated. Approximately, 95,847,818 raw pair-end reads were generated, resulting in a total of 70,879,904 clean reads with a length of 90 bp. We further mapped the clean reads to *Brassica rapa* genome and found that the clean reads were mainly distributed in the coding sequence region (Figure 1A), which indicated that transcriptome sequencing had good quality. All the clean reads were *de novo* assembled by Trinity programs (Grabherr et al., 2011), which generated a total of 68,086 unigenes with an average length of 576 bp and an N_50_ of 773 bp (Figure 1B). Among the unigenes, the shortest and longest unigenes are 201 and 7884 bp, respectively. Moreover, 34,090 unigenes were within the 200–400 bp, and 24,262 unigenes were within the 400–1000 bp. In the meanwhile, we observed that part of unigenes (9734) were over 1000 bp, which is very helpful to further annotation and functional analysis of unigenes. Meanwhile, we made a comparison of *de novo* assembly between the earlier and current sequencing data from leaf tissues. A better *de novo* assembly was obtained in current sequencing data and the results are shown in Supplementary Table S1.

In order to assess the quality of *de novo* assembly of *R. sativus*, the clean reads were mapped to the assembled unigenes. We observed that 45,296,360 (64%) reads were mapped to the assembled unigenes. Among the mapped reads, 29,997,587 (66.23%) reads could uniquely map to the unigenes, while 15,298,773 (33.77%) reads could map to multiple locations on unigenes. In addition, to estimate the level of transcripts coverage of *R. sativus*, indirect evaluation was performed as described (Zhang et al., 2013). The result showed that the *de novo* assembly generated in this study was estimated to cover 47.2% of the *R. sativus* transcriptome. Given that only one tissue (leaf) was used for sequencing and *de novo* assembly, 47.2% trasncriptome coverage should be appropriate for the annotation and functional research in the future.

For further homology analysis, we compared the assembled leaf unigenes with the CDS sequences of *A. thaliana*, *B. rapa* and *Thellungiella halophila*. Among the 68,086 unigenes, a total of 53,384, 56,695, and 54,003 sequences were mapped to the CDS region in *A. thaliana*, *B. rapa*, and *T. halophila*, respectively. The identity of the significant hits against *A. thaliana* ranged from 77.65 to 100%, with an average value of 88.61% and an N_50_-value of 88.67%. The identity of the significant hits against *B. rapa* varied from 77.97 to 100%, with an average value of 93.02% and an N_50_-value of 93.6%. While the identity of the hits against *T. halophila* ranged from 77.54 to 100%, with an average value of 89.71% and an N_50_-value of 89.94%. In the meanwhile, we also compared the leaf unigenes with the mRNA sequences of *A. thaliana*, *B. rapa*, and *T. halophila*. Almost similar results were obtained as shown in Supplementary Table S2. Collectively, the results indicated that the leaf transcriptome of *R. sativus* has high similarity with those of other *Brassicaceae* species.

The assembled leaf unigenes were annotated by using BLASTx against the NCBI non-redundant protein (Nr) database, the Swiss-Prot protein database, Pfam database, Nt database and Trembl database with an *E*-value cutoff of 1 e^−5^. The mapping rates of the annotated unigenes were between 52 and 87% (Table 1). Among the 68,086 unigenes, 52,677 (77.37%) had at least one significant match with an *E*-value less than 1 e^−5^ against the Nr database. The mapping rates of unigenes against the Swissprot and Trembl protein databases were 52.75 and 77.15%, respectively. The results indicated that most of the unigenes are protein coding genes. More than 90% of the unigenes over 700 bp in length had homology matches, whereas only less than 60% of the unigenes shorter than 300 bp had significant matches (Figure 1C). These short unigenes may lack a known conserved functional domain or represent non-coding RNAs so that they had no significant matches. Alternatively, these unigenes may contain a known protein domain but too short to display sequence matches, resulting in false-negative results. Moreover, the genomic and transcriptomic information is limited for *R. sativus*, and many *R. sativus* lineage-specific genes might not be contained in the database. Based on the annotation results of the Nr database, the *E*-value distribution analysis showed that 65.77% of the matched sequences had strong homology with the *E*-value < 1 e^−30^, and 46.85% of the matched sequences showed strong homology with the *E*-value < 1 e^−45^, whereas only 34.23% of the matched sequences had high similarity with the *E*-value from 1 e^−30^ to 1 e^−5^ (Figure 1D). The similarity distribution analysis revealed that 65.72% of the mapped sequences had a similarity higher than 80%, whereas 34.28% of mapped sequences had a similarity ranging from 23 to 80% (Figure 1E). The species distribution analysis revealed that leaf tissue in *R. sativus* has a number of homologous sequences in many plant species (Figure 1F). For instance, *Arabidopsis lyrata genes* have the highest similarity with *R. sativus* unigenes among the various plant species (46.13%), followed by *A. thaliana* (37.61%), *Eutrema halophilum* (3.89%), *B. rapa* (2.33%), *Brassica napus* (1.94%), and *Brassica oleracea* (1.77%). This result suggested that the genome of *A. lyrata* or *A. thaliana* can be used as a reference for the transcriptome analysis of leaf tissue in *R. sativus*.

**Table 1:** **Blast results of the assembled leaf unigenes of *R. sativus***.

**Database**	**Total unigenes**	**Total annotated unigenes**	**Mapped unigenes**
Nr			52,677(77.37%)
Nt			59,115(86.82%)
Pfam	68,086	60,685	43,447(63.80%)
SwissProt			35,915(52.75%)
Trembl			52,525(77.15%)

In order to validate redundancy of the sequences with publicly available data, we compared the leaf unigenes with the *R. sativus* unigene database on the Genoscope (http://www.ncbi.nlm.nih.gov/unigene/) using blastn with an *E*-value cutoff of 1 e^−10^. Among the 68,086 unigenes, 32,255 (47.37%) had significant similarity to *R. sativus* unigene database. Meanwhile, we found that a total of 35,831 (52.63%) unigenes had no significant match, which indicated that they were probably the non-redundant unigenes (Zhang et al., 2012b). The length of the non-redundant unigenes varied from 201 to 3594 bp, with an average value of 442 bp and an N_50_-value of 320 bp (Figure 1G). In addition, the leaf unigenes were blasted against the *R. sativus* mRNA database with an *E*-value cutoff of 10^−10^ for identifying novel transcribed sequences. A total of 15,729 (23.11%) unigenes had no match with the *R. sativus* mRNA database, and were considered potential novel transcribed sequences. The length of the novel transcribed sequences varied from 201 to 3044 bp, with an average length of 368 bp and an N_50_-value of 283 bp (Figure 1G).

Given that there are available transcriptome sequencing data of root tissue in *R. sativus* (Wang et al., 2013a), we thus downloaded root data (SRX256970 and SRX263753) and merged the clean reads of leaf and root sequencing data for *de novo* assembly using Trinity (Grabherr et al., 2011) and Oases programs (Schulz and Zerbino, 2010). The Trinity assembly generated a total of 103,222 unigenes with an average length of 786 bp and an N_50_ of 1250 bp (Supplementary Table S3). Among the unigenes, the shortest and longest unigenes are 201 and 15,670 bp, respectively. Statistical analysis showed that 44,479 unigenes were within the 200–400 bp, and 32,168 unigenes were within the 400–1000 bp. Moreover, we observed that 26,575 unigenes were over 1000 bp. Meanwhile, the Oases assembly generated a total of 106,874 unigenes with an average length of 1108 bp and an N50 of 1770 bp. The assembled unigenes generated from Trinity and Oases were deposited in NCBI TSA database with accession number GBZO01 and GBZP01, respectively. Also, the comparison analysis between Trinity and Oases assembly were summarized in Table 2. As the unigenes with longer average length were generated using Oases than Trinity, we thus used Oases-assembled unigenes for further annotation and downstream analyses. The Oases-assembled unigenes were annotated by using BLASTx against the NCBI non-redundant protein (Nr) database, COG database, Swiss-Prot protein database, and Pfam database with an *E*-value cutoff of 1 e^−5^. The mapping rates of the annotated unigenes were listed in Table 3.

**Table 2:** ***De novo* assembly analysis using Trinity and Oases**.

**Assembly**	**Trinity**	**Oases**
Number of clean reads	125,591,478	125,591,478
Total unigenes generated	103,222	106,874
N_50_ length (bp)	1250	1770
Average unigene length (bp)	786	1108
Length of longest unigene	7884	15,647

**Table 3:** **Blast results of Oases-assembled *R. sativus* unigenes**.

**Database**	**Total unigenes**	**Mapped unigenes**
Nr	106,874	76,520 (71.6%)
COG		42,171 (39.5%)
Pfam		62,843 (58.8%)
SwissProt		62,497 (58.5%)

### Functional classification of *R. sativus* unigenes

**Figure 2:**
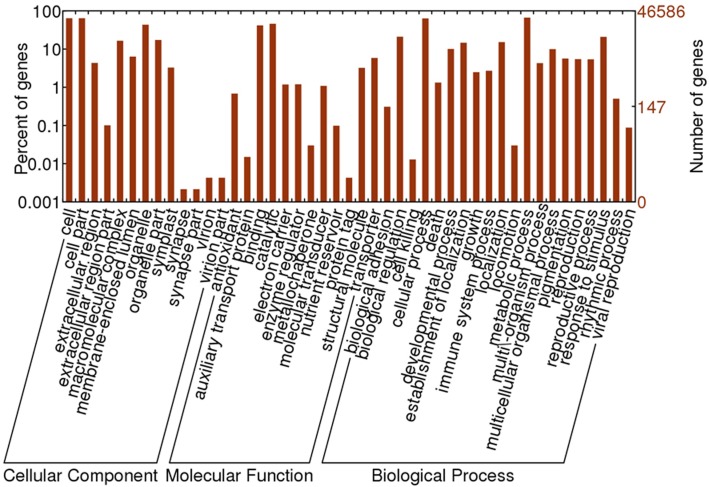
**The GO annotation of *R. sativus* transcriptome**.

Based on sequence homology, 46,586 Oases-assembled *R. sativus* unigenes were assigned with GO terms. The annotated GO terms were classified into 45 functional groups, which were distributed under the three main categories including molecular function, biological process and cellular component (Figure 2). Within the molecular function category, “catalytic,” “binding” and “transporter” were top three GO terms. Among the biological process, “cellular process,” “metabolic process,” “response to stimulus,” “biological regulation,” “localization,” and “establishment of localization” were major GO terms. In the cellular component, “cell,” “cell part,” “organelle,” and “organelle part” were mainly enriched. Furthermore, only a few unigenes were classified in terms of “synapse,” “synapse part,” “virion,” “virion part,” “protein tag,” “cell killing,” “auxiliary transport protein activity,” and “metallochaperone activity.”

We further performed a systematic analysis of high-level gene function by assigning *R. sativus* unigenes to the biochemical pathways in the KEGG database. All Oases-assembled unigenes were compared with the KEGG database to identify the biological pathways. As a result, a total of 42,181 unigenes were annotated in KEGG database, which were associated with 240 KEGG pathways (Supplementary File 1). The top 10 enriched pathways were “Glycolysis/Gluconeogenesis” (660 sequences), “Citrate cycle (TCA cycle)” (331 sequences), “Pentose phosphate pathway” (299 sequences), “Pentose and glucuronate interconversions” (491 sequences), “Fructose and mannose metabolism” (278 sequences), “Galactose metabolism” (314 sequences), “Ascorbate and aldarate metabolism” (346 sequences), “Fatty acid biosynthesis” (122 sequences), “Fatty acid elongation in mitochondria” (11 sequences), “Fatty acid metabolism” (258 sequences). Moreover, KEGG analysis showed that 364 unigenes were involved in the alkaloid biosynthesis. As alkaloids are very necessary for defending against pathogens, the result confirmed that *R. sativus* is an important source of medicinal compounds (Wang et al., 2002).

### Analysis of the assembled transcripts in leaf and root tissues

To investigate which unigenes were unique in the leaf tissue, the leaf unigenes (Table 1) were compared to the unigenes of root tissue of *R. sativus* available in SRA database (Wang et al., 2013a) by using blastx with an *E*-value cutoff of 1 e^−5^. As a result, a total of 60,633 (89%) leaf unigenes had perfect match to root unigenes, which indicated that the transcripts of root and leaf tissue of *R. sativus* are very similar. There were 7453 “leaf-specific” unigenes showing no significant homology to the transcripts of root tissue. In the meanwhile, KEGG analysis showed these unigenes were assigned to 71 pathways (Supplementary File 2). The top five pathways (ranked by *p*-value) included “Ribosome” (ko03010), “Nitrogen metabolism” (ko00910), “Carbon fixation in photosynthetic organisms” (ko00710), “Photosynthesis” (ko00195) and “Glycolysis/Gluconeogenesis” (ko00010). Ribosome biogenesis is one of the most important cellular processes and makes up the key component to regulate overall protein synthesis and cell growth (Deisenroth and Zhang, 2011). Nitrogen metabolism, Carbon fixation in photosynthetic organisms, and Glycolysis/Gluconeogenesis are all associated with photosynthesis. Photosynthesis is a necessary process used by plants to convert light energy into chemical energy and carbohydrates that can be released to fuel the organisms' activities. Collectively, the results indicated that these leaf-specific unigenes are mainly involved in the photosynthesis pathway. In addition, 15,043 root-specific unigenes were identified. KEGG analysis revealed these root-specific genes were assigned to 96 pathways (Supplementary File 3). The top five pathways (ranked by *p*-value) included “Phenylalanine metabolism” (ko00360), “Methane metabolism” (ko00680), “Diterpenoid biosynthesis” (ko00904), “Phenylpropanoid biosynthesis” (ko00940), and “Biosynthesis of phenylpropanoids” (ko01061). Among these pathways, phenylalanine metabolism has been proved to play an important role in root growth (Herrig et al., 2002), while methane is metabolized principally by methanotrophs and methanogens in the global carbon cycle, which is helpful for plants to obtain energy for growth (Herrig et al., 2002).

**Figure 3:**
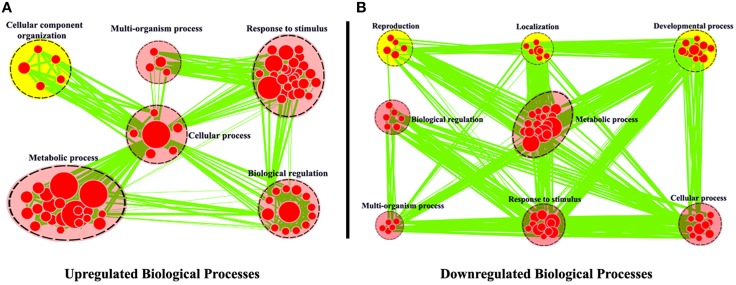
**Biological Process analysis of differentially expressed genes between leaf and root tissues**. GO modules enriched with up-regulated DEGs **(A)** and down-regulated DEGs **(B)** were visualized by the Enrichment Map in Cytoscape. The red and yellow circles indicate the common and different biological processes between up-regulated and down-regulated DEGs, respectively.

The expression levels of root unigenes were defined as control to identify the differentially expressed genes (DEG) between leaf and root tissues in *R. sativus*, the gene expression levels were calculated using the RPKM method (Reads per Kb per Million mapped reads). A total of 3563 significantly differentially expressed genes (*P* = 0.05) were identified, including 2335 up-regulated and 1228 down-regulated genes. To achieve a functional annotation of differentially expressed genes, GO classifications were assigned to the differentially expressed genes by using Cytoscape Enrichment Map (http://www.cytoscape.org/). As shown in Figure 3, 2613 DEGs were assigned with Biological Process terms. Within the biological process category, the common GO terms between up-regulated genes and down-regulated genes were “Metabolic process,” “Response to stimulus,” “Cellular process,” “Multi-organism process,” and “Biological regulation.” In the meanwhile, we observed that some DEGs were clustered in different Go terms. For example, 95 genes were up-regulated in leaf tissue and significantly enriched in “Cellular component organization.” Nevertheless, we observed that 172 genes were up-regulated in root tissue and significantly enriched in “Developmental process,” 63 genes were up-regulated in root tissue and significantly enriched in “Reproduction” and 96 genes were up-regulated in root tissue and significantly enriched in “Localization.” We further examined gene functions in these different GO terms by KEGG analysis. The result showed that many genes in “Cellular component organization,” such as AT5G66570 (Oxygen-evolving enhancer protein), AT2G47910 (Chlororespiratory reduction 6) and etc., are involved in photosynthesis pathway in leaf tissue (Supplementary Figure 1), which supports the feasibility of GO classifications in Figure 3. In the meanwhile, we also examined the functions of genes in “Developmental process,” “Reproduction,” and “Localization” by KEGG analysis. The results showed that many genes up-regulated in root tissue are involved in spliceosome pathway, tryptophan metabolism pathway and protein export pathway. Taken together, these KEGG results explain the basis of GO classifications of such DEGs between leaf and root tissues and are helpful for functional genomics study of these unigenes in *R. sativus*.

### SSR analysis of the transcripts in *R. sativus*

**Figure 4:**
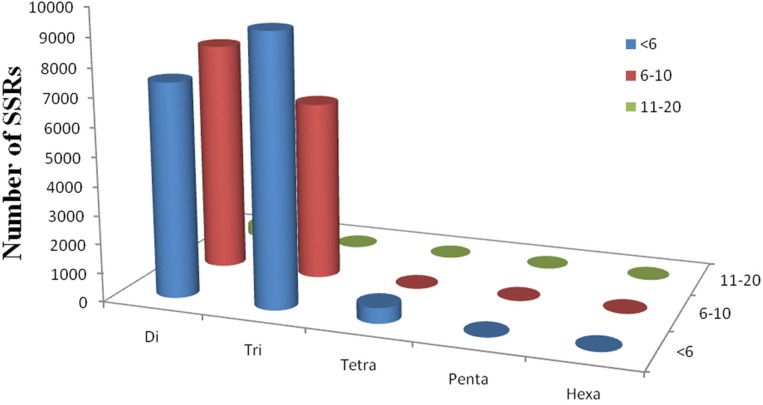
**The SSR mining results in *R. sativus***. The profiles of different SSR types in *R. sativus*.

Simple sequence repeats (SSRs) have been the most popular molecular-marker for various applications, such as quantitative trait loci (QTL) exploration, genome mapping, and genetic analysis (Li et al., 2002, 2004; Sharopova et al., 2002; Xu et al., 2011; Yu et al., 2011). In this study, we predicted potential SSRs in the assembled unigenes of *R. sativus*. A total of 31,875 potential SSRs were identified in 13,469 unigenes, of which 10,143 unigenes contained more than one SSR. A comprehensive analysis was performed to describe the type, frequency and distribution of all the potential SSRs. On average, one SSR could be found every 1.87 kb in the unigenes. The SSRs included 15,745 (49.39%) dinucleotide motifs, 15,555 (48.80%) trinucleotide motifs, 552 (1.73%) tetranucleotide motifs, 13 (0.04%) pentanucleotide motifs, and 10 (0.03%) hexanucleotide motifs (Figure 4). The number distribution of repeat units in all SSRs was also summarized (Table 4). The result showed that the repeat number of most SSRs was no more than 10, and no SSRs with more than 20 repeat sequences were observed. In addition, the repeat number and the total repeat length (= type number ^*^ type length ^*^ type average repeat number) of each SSR type were analyzed (Supplementary File 4). For most dinucleotide type, the repeat number was distributed between 5 and 11, with an average value of 6.31. The average repeat number of trinucleotide and tetranucleotide type was 5.49 and 5.06, respectively. While all the repeat number of pentanucleotide and hexanucleotide type was only five. Collectively, a total of 31,875 potential SSRs were identified from transcriptomic data of *R. sativus*, which is helpful for different aspects of agronomic research. However, transcriptome-derived SSR identification is mainly based on CDS information, which led to the omission of a fairly large number of SSRs in the intronic and non-coding regions in comparison with genome sequence-derived SSR identification.

**Table 4:** **SSRs distribution in the unigenes of *R. sativus***.

**Type**	**Repeats number**			**Total**
	**<6**	**6–10**	**10–20**	
Di-nucleotide	7431	7959	355	15,745
Tri-nucleotide	9373	6182	0	15,555
Tetra-nucleotide	540	12	0	552
Penta-nucleotide	13	0	0	13
Hexa-nucleotide	10	0	0	10

### Transcription factors identification and network analysis of the transcripts

**Figure 5:**
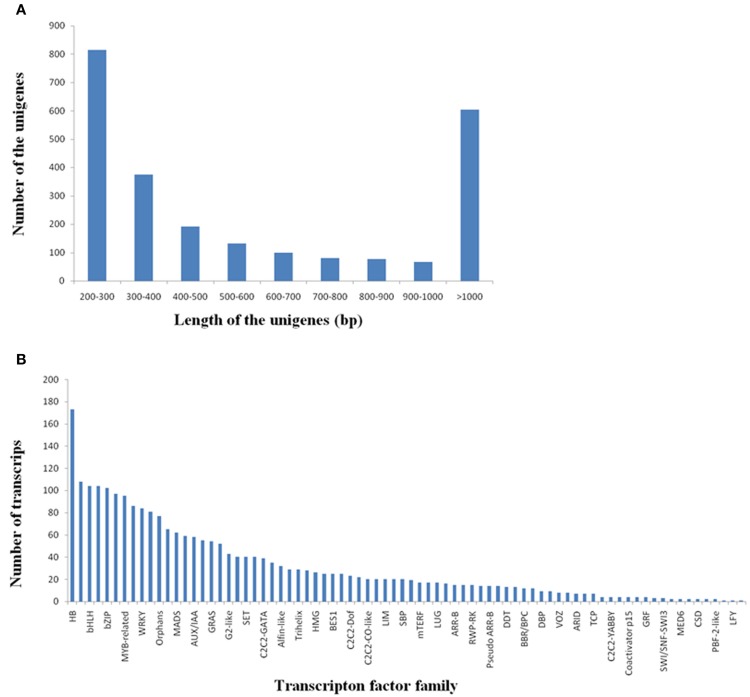
**Discovery of the transcripts encoding transcription factors in *R. sativus*. (A)** Length distribution of potential transcription factor genes. **(B)** Transcripts distribution in various transcription factor families.

Transcription factors (TFs) play important roles in multiple biological processes. Here, we identified a total of 2440 potential TF unigenes by comparing *R. sativus* unigenes with the plant transcription factor database. The length of these TF unigenes varied from 200 to 7043 bp, with an average value of 737 bp and an N_50_-value of 415 bp. The 200–300 bp class was the most enriched in total sequence number (33.37%), followed by >1000 bp (24.76%), 300–400 bp (15.33%), 400–500 bp (7.83%), 500–600 bp (5.41%), 600–700 bp (4.10%), 700–800 bp (3.32%), 800–900 bp class (3.16%), and 900–1000 bp (2.75%) (Figure 5A). The potential TFs were distributed in 78 families, such as HB, MYB, bHLH, C3H, bZIP, NAC, WRKY, and so on (Figure 5B). Among these TF gene families, HB, MYB and bHLH were the most abundant TF families (Supplementary File 5). It was reported that HBs may be involved in cell differentiation and control of cell growth, as well as patterning of diverse organisms. (Kappen, 2000). MYB proteins constitute a diverse class of DNA-binding proteins, which associate with regulation of secondary metabolism, control of cellular morphogenesis and regulation of meristem formation and the cell cycle (Jin and Martin, 1999). For bHLH (basic helix-loop-helix) family, its members were reported to participate in controlling cell proliferation and development of specific cell lineages (Heim et al., 2003).

**Figure 6:**
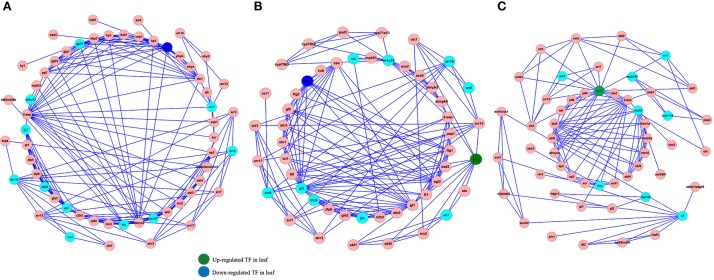
**Construction of TFs-based regulation network by Cytoscape software**. Sky blue and red circles represent identified TFs and genes involved in the network **(A–C)**, respectively. The blue and green circles represent up-regulated and down-rugulated TFs in leaf.

Transcription factors play an important role in regulating almost each aspect of the organism's life metabolism. Nevertheless, understanding how TF-based regulation network eventually affect the phenotypes remains still elusive. In order to analyze the interaction between TFs and their gene targets, we used the Cytoscape software to construct a TF-based regulation network with 142 nodes and 460 edges. As shown in Figure 6, a total of 21TFs, such as SPA1, EIN3, WRKY33, and etc., were involved in the complicated regulation network. Network analysis showed that SPA1 interacts with three targets including ATMYC2, CAB1, and COP1. For example, it was reported that ATMYC2 and SPA1 act redundantly to suppress photomorphogenic growth in *A. thaliana* (Gangappa et al., 2010). We also observed that EIN3 interacts with EIL1, EIN2, EBF1, EBF2, AP2, ARR3, ARR5, and ARR15. Among these target genes, EIL1, EIN2, EBF1, EBF2, and AP2 were all linked with ethylene mediated signaling pathway. Accordingly, ARR3, ARR5, and ARR15 were all involved in the regulation process of circadian rhythm, red light signaling pathway and etc.

### Enzyme detection and characterization of *R. sativus*

**Figure 7:**
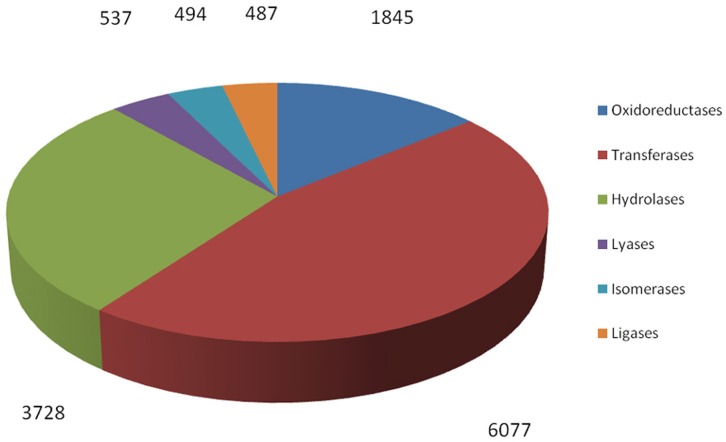
**Functional characterization and classification of potential enzyme genes in the transcripts of *R. sativus***.

By searching against the available database, a total of 12,238 *R. sativus* unigenes were annotated with 13,168 enzyme codes. The annotated enzyme codes included six classes: Oxidoreductases (1845 sequences), Transferases (6077 sequences), Hydrolases (3728 sequences), Lyases (537 sequences), Isomerases (494 sequences), and Ligases (487 sequences) (Figure 7). Furthermore, 967 enzyme types were detected. The most abundant enzyme types were non-specific serine/threonine protein kinase (2258 sequences), phosphoprotein phosphatase (491 sequences) and RNA helicase (305 sequences), respectively (Supplementary File 6). These enzymes were associated with various biological processes. For instance, through the phosphorylation of the serine or threonine residues in their substrate, serine/threonine protein kinases play an important role in many cellular processes such as post-translational modification and are potential drug targets in human cancers, inflammation, and metabolic diseases (Mumby and Walter, 1993). In contrast, phosphoprotein phosphatases usually remove phosphate groups from the substrates and are involved in many cellular processes including metabolism, gene transcription and translation (Shi, 2009). Collectively, we identified a bunch of enzyme codes in the transcripts *of R. sativus* by searching their unigenes against the databases. As enzymes are a type of large biological molecules responsible for thousands of metabolic processes that sustain life, the identification of important enzyme codes might provide key clues to reveal some important functional pathways and metabolic activities between leaf tissue and root tissue in *R. sativus*. In the meanwhile, we ran RepeatMasker to identify repetitive elements in leaf and root transcriptome of *R. sativus*. The generated results were listed in Supplementary Table S4.

## Conclusions

The transcriptome of leaf tissue in *R. sativus* was sequenced by Illumina Solexa technology. Approximately 70 million clean pair-end reads were obtained and used for the *de novo* assembly by Trinity program, which generated 68,086 unigenes with an average length of 576 bp. In the meanwhile, we merged leaf sequencing data with existing root sequencing data and obtained better *de novo* assembly of *R. sativus* using Oases program. Accordingly, potential simple sequence repeats (SSRs), transcription factors (TFs) and enzyme codes were identified in *R. sativus*. Additionally, we detected a total of 3563 DEGs (*P* = 0.05) and tissue-specific biological processes between leaf and root tissues. Furthermore, a TFs-based regulation network was constructed using Cytoscape software. Taken together, this study not only provides a critical genomic resource of *R. sativus*, but also paves a way for gene expression analysis, functional genomics and even proteomics research on *R. sativus* in the future.

## Database linking

The sequencing data of leaf and root tissues supporting the results of this study were deposited in NCBI Sequence Read Archive (SRA) Sequence Database with accession number SRP038118 and SRX256970.

## Author contributions

LM and ZY conceived and designed the experiments and contributed the reagents. WG and ZL analyzed the data and wrote the manuscript. YY performed the RNA isolation experiment. WJ provided the *R. sativus* materials and participated in the design of the study. YL helped to analyze the data and draft the manuscript. LM coordinated the study and revised the manuscript. All authors read and approved the final manuscript.

### Conflict of interest statement

The authors declare that the research was conducted in the absence of any commercial or financial relationships that could be construed as a potential conflict of interest.
