# Supplementary material for: Sequencing, de novo assembly and comparative analysis of Raphanus sativus transcriptome
Source: Front Plant Sci. 2015 Apr 1;6:198. doi: 10.3389/fpls.2015.00198 (PMC4428447; doi:10.3389/fpls.2015.00198)
Supplement: Supplementary file 1 [file Table1.DOCX]

| Supplementary Table S1. Comparison of leaf transcriptome assembly with previous data |
| --- |
| Assembly Previous data Current data |
|  |
| Number of raw reads 27,976,916 95,847,818 |
| Number of used reads 21,978,870 70,879,904 |
| Total Unigenes generated 28,410 68,086 |
| N_50_ length (bp) 422 773 |
| Average Unigene length (bp) 394 576 |
