# Supplementary material for: Sequencing, de novo assembly and comparative analysis of Raphanus sativus transcriptome
Source: Front Plant Sci. 2015 Apr 1;6:198. doi: 10.3389/fpls.2015.00198 (PMC4428447; doi:10.3389/fpls.2015.00198)
Supplement: Supplementary file 2 [file Table2.DOCX]

| Supplementary Table S2: Homology analysis of assembled leaf unigenes with different species | | |
| --- | --- | --- |
| Species | **Mapped to CDS** | **Mapped to mRNA** |
| *A. thaliana* | 53384 (78.41%) | 54154 (79.54) |
| *B. rapa* | 56695 (83.27%) | 59514 (87.41%) |
| *T. halophila* | 54003 (79.32%) | 54933 (80.68%) |
