# Supplementary material for: Sequencing, de novo assembly and comparative analysis of Raphanus sativus transcriptome
Source: Front Plant Sci. 2015 Apr 1;6:198. doi: 10.3389/fpls.2015.00198 (PMC4428447; doi:10.3389/fpls.2015.00198)
Supplement: Supplementary file 3 [file Table3.DOCX]

| Supplementary Table S3. Comparison of transcriptome assembly (Leaf vs Leaf and Root) |
| --- |
| Assembly Leaf Leaf and Root |
|  |
| Number of clean reads 70,879,904 125, 591, 478 |
| Total Unigenes generated 68,086 103, 222 |
| N_50_ length (bp) 773 1,250 |
| Average Unigene length (bp) 576 786 |
