# Supplementary material for: Sequencing, de novo assembly and comparative analysis of Raphanus sativus transcriptome
Source: Front Plant Sci. 2015 Apr 1;6:198. doi: 10.3389/fpls.2015.00198 (PMC4428447; doi:10.3389/fpls.2015.00198)
Supplement: Supplementary file 4 [file Table4.DOCX]

| **Supplementary Table S5:Identification of transposon in *R.sativus*** | |
| --- | --- |
| **SINEs** | 14 |
| **LINEs** | 107 |
| **LTR elements** | 43 |
| **DNA elements** | 30 |
