# Supplementary figures and images for: Sequencing, de novo assembly and comparative analysis of Raphanus sativus transcriptome
Source: Front Plant Sci. 2015 Apr 1;6:198. doi: 10.3389/fpls.2015.00198 (PMC4428447; doi:10.3389/fpls.2015.00198)

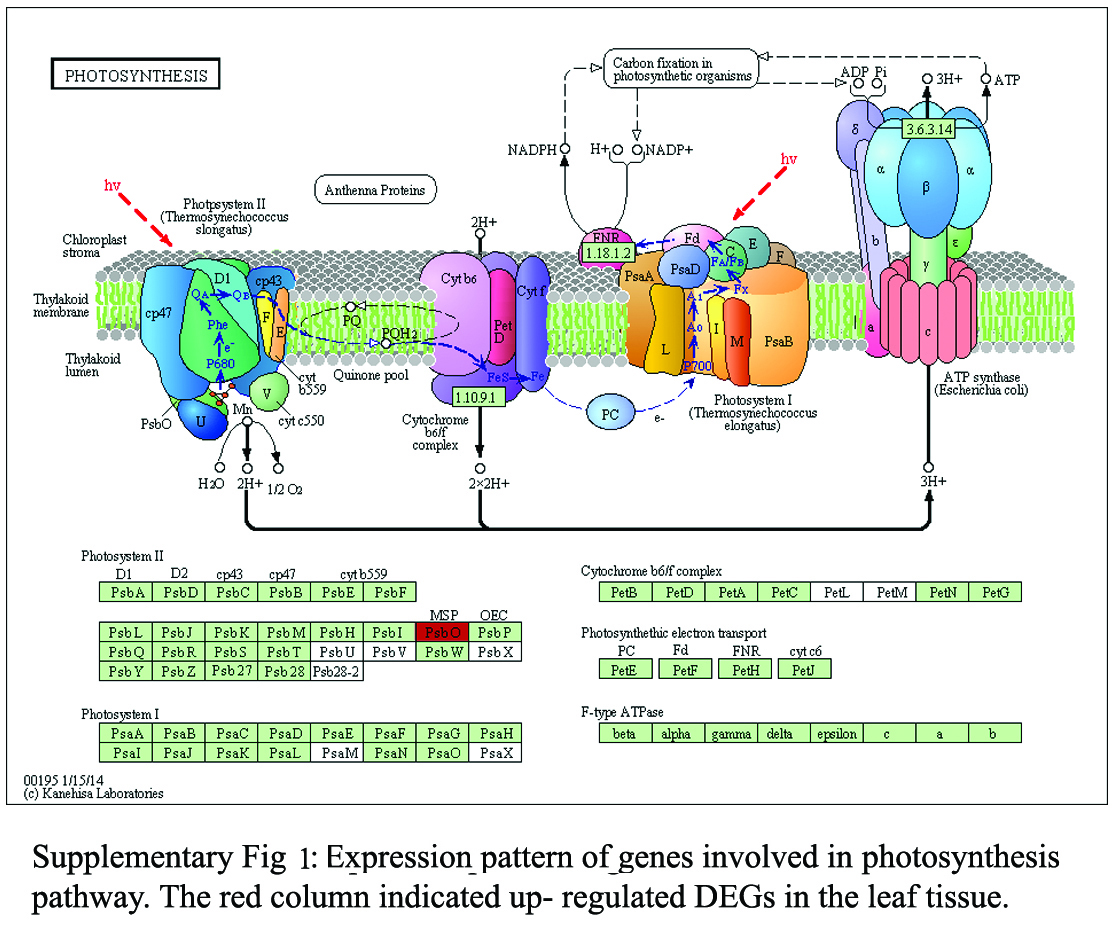

Supplement: Supplementary file 11 [file Image1.TIF]
